# Integrating PEESS, EREFS, and endoscopic ultrasonography in the multimodal management of pediatric eosinophilic esophagitis: A 4-year retrospective institutional experience

**DOI:** 10.1097/MD.0000000000043803

**Published:** 2025-08-08

**Authors:** Gül Çirkin, Yunus Güler, Özlem Gülpinar Aydin, Özge Atay, Özge Kangalli Boyacioğlu, Suna Asilsoy, Nevin Uzuner, Yeşim Öztürk

**Affiliations:** a Department of Pediatrics, Dokuz Eylul University Faculty of Medicine, Division of Pediatric Gastroenterology, Hepatology and Nutrition, Izmir, Turkey; b Department of Pediatrics, Dokuz Eylul University School of Medicine, Division of Pediatric Allergy Immunology, Izmir, Turkey.

**Keywords:** endoscopic ultrasonography, eosinophilic esophagitis, EREFS, histological remission, pediatrics, PEESS, targeted therapy

## Abstract

Pediatric eosinophilic esophagitis (EoE) is a chronic, immune-mediated disorder defined by esophageal dysfunction and eosinophilic infiltration of the esophageal mucosa. Its incidence is increasing, particularly among children. However, assessing disease severity and monitoring treatment response over time remain challenging. Conventional histological methods often fail to capture ongoing inflammation or predict symptom recurrence. This study aimed to evaluate the utility of a multimodal approach incorporating clinical symptom scoring (Pediatric Eosinophilic Esophagitis Symptom Score [PEESS]), endoscopic findings (Eosinophilic Esophagitis Reference Score [EREFS]), and subepithelial imaging (endoscopic ultrasonography [EUS]) for the diagnosis, treatment monitoring, and long-term follow-up of pediatric EoE. We conducted a retrospective review of 23 pediatric patients diagnosed with EoE between 2018 and 2022 at a tertiary care center. Diagnosis was based on clinical presentation, endoscopic appearance, and histological confirmation (≥15 eosinophils/high-power field). All patients received a combination of proton pump inhibitors, topical corticosteroids, and personalized elimination diets. PEESS and EREFS scores were assessed before and after treatment. EUS was performed in a subset of patients to evaluate esophageal wall changes. The median age at diagnosis was 11 years. Atopic comorbidities were identified in 82.6% of patients. Post-treatment, both PEESS and EREFS scores showed significant improvements. Histological remission was achieved in 95.6% of cases. Among the 8 patients who underwent EUS, persistent esophageal wall thickening was observed despite mucosal healing, suggesting ongoing subepithelial inflammation. Long-term dietary management without steroids was successful in a subset of patients after initial remission. PEESS and EREFS are valuable, complementary tools for assessing treatment response in pediatric EoE. EUS provides additional insights into residual disease activity beyond mucosal healing. This multimodal evaluation may facilitate personalized, less-invasive, and more effective long-term management of pediatric EoE.

## 1. Background

Eosinophilic esophagitis (EoE) is a long-term immune-related condition defined by clinical symptoms linked to esophageal dysfunction and histologically marked by eosinophilic inflammation in the esophagus.^[1]^ Over the past few decades, the frequency and occurrence of EoE have increased, making it the primary cause of dysphagia in children and young adults^.[2–4]^

The range of differential diagnoses for EoE is wide and may include gastroesophageal reflux disease, parasitic and fungal infections, inflammatory bowel conditions, allergic vasculitis, connective tissue disorders, and various other conditions related to esophageal eosinophilia.^[5–7]^

The diagnosis of EoE is based on symptoms, endoscopic appearance, and histological findings. Common endoscopic features include a thickened, occasionally pale esophageal lining accompanied by rings, linear furrows, and white plaques, with esophageal narrowing being observed less frequently. A normal endoscopic appearance of the esophagus does not rule out EoE. To optimize diagnostic sensitivity, it is recommended to obtain a minimum of 2 to 4 biopsy specimens from both the proximal and distal regions of the esophagus, regardless of the endoscopic presentation.^[8]^ The primary histological features consist of significant eosinophil accumulation within the esophageal mucosa, basal zone hyperplasia, lamina propria fibrosis, and occasionally eosinophilic microabscesses, which are often distributed throughout the esophagus.^[9]^

An additional diagnostic method, endoscopic ultrasonography (EUS), demonstrates thickening of the deeper layers of the esophagus.

Fibrosis of the mucosal and submucosal layers, together with hypertrophy of the smooth muscle, most likely leads to reduced esophageal flexibility and may play a role in dysphagia symptoms even in the absence of a visible stricture.^[10]^

Disease therapy includes dietary and pharmaceutical interventions, each of which has advantages and disadvantages. The aim of this study was to review and present the clinical, laboratory, and therapeutic procedures of patients diagnosed with EoE in our center over a 4-year period.

## 2. Material and methods

This study was approved by the Ethics Committee of Dokuz Eylul University (approval number: 2022/17-12; date: May 11, 2021). We retrospectively reviewed the medical records of patients diagnosed with EoE at the Division of Pediatric Gastroenterology, Hepatology, and Nutrition of the Dokuz Eylul University between 2018 and 2022. The diagnosis of EoE was established based on histological findings, with a threshold of > 15 eosinophils per high-power field in biopsies obtained from both the upper and lower esophagus.^[8,11]^ Additionally, biopsies were collected from the stomach and duodenum; however, no significant eosinophilic infiltration was observed in these regions. In cases where endoscopy and EUS were performed, informed consent was obtained from the patients’ families prior to the procedure.

Two different sizes of video gastroscopes were used for endoscopic procedures (Video Gastroscope FUJINON/e.g.-530WR and Pediatric Video Gastroscope FUJINON/e.g.-530 NP SUPER SLIM). During endoscopic evaluation, cases with normal-appearing mucosa and histologically confirmed EoE were incidentally identified. To enhance mucosal visualization, EUS imaging was performed in selected patients during follow-up. However, this method has not been applied in all patients. In total, 8 patients underwent EUS based on clinical indication and endoscopic findings. Radial endoscopic ultrasound imaging was performed using the Radial Endoscopic Ultrasonography FUJINON e.g.-530UT2 system.

The Eosinophilic Esophagitis Reference Score (EREFS) was used to assess disease activity and monitor follow-up.^[11]^

Vomiting severity was categorized based on criteria defined in the literature and retrospectively analyzed through a review of medical records, including anamnesis and symptom assessments. In addition, clinical symptom severity was assessed using a standardized tool, the Pediatric Eosinophilic Esophagitis Symptom Score (PEESS), by the same evaluator before and after each endoscopy.^[12]^

Patient data, including body weight, height, body mass index percentiles, laboratory findings (plasma eosinophilia percentage, specific immunoglobulin E [IgE]), medical history, endoscopy indications, esophagogastroduodenal radiographs, and EUS images, were extracted from the hospital’s electronic medical record system and retrospectively analyzed.

Food allergens were evaluated by pediatric allergists using the IVD-approved CE-certified EUROLINE immunoblot strip “Turkey Food Panel” (Cat. No: DP 3420-1601-11-E; Euroimmun, Luebeck, Germany). The presence of specific IgE was also assessed.

The recommended proton pump inhibitor (PPI) dose for EoE was 1 mg/kg/day. Budesonide was given as an oral suspension with honey (220 µg for children aged 1–10 years and 440 µg twice daily for those aged ≥ 11 years). Treatment regimens were adjusted based on histological findings, with reassessments performed via endoscopic biopsies at 3 to 6-month intervals^.[13]^

All patients initially received a single daily dose of PPI. Steroid and PPI therapies were initiated simultaneously in all patients. Steroid therapy was administered twice daily at the beginning of treatment and was gradually tapered and discontinued once eosinophils were no longer detected in histological assessments of endoscopic biopsies. Following discontinuation of steroid therapy, the patients were managed exclusively with a specific elimination diet for long-term maintenance.

### 2.1. Statistical analysis

Statistical analyses were performed using the IBM SPSS Statistics for Windows (version 19.0; SPSS Inc., Chicago, IL). The Shapiro–Wilk test was used to assess the normality of continuous variables. Data with normal distribution are expressed as mean ± standard deviation, whereas non-normally distributed data are presented as median and interquartile range. Categorical variables are summarized as frequencies and percentages. Parametric tests (*t* test) were used for normally distributed variables, while non-parametric tests (Mann–Whitney *U* test) were applied otherwise. Categorical variables were analyzed using chi-square or Fisher exact test as appropriate.

To compare paired, non-normally distributed variables, such as pre- and post-treatment PEESS and EREFS scores, the Wilcoxon signed-rank test was used. Statistical significance was set at *P* < .05. The results are reported as median (minimum–maximum) or mean ± standard deviation, as appropriate.

## 3. Results

### 3.1. Study population and clinical characteristics

This study included 23 pediatric patients diagnosed with EoE based on the esophageal biopsy findings. The mean patient age was 11 years. A detailed assessment of atopic history revealed asthma in 13 cases, eczema in 2, allergic rhinitis in 2, pet allergy in 1, and food allergy in 1. No allergic history was identified in 4 patients.

### 3.2. Endoscopic indications and symptomatology

The decision to perform endoscopy was based on clinical presentation. Symptom severity varied across patients; gastrointestinal reflux disease was observed in 4 cases, dysphagia in 9, and vomiting in 10 (Table 1).

**Table 1:** Characteristics of 23 pediatric cases with EoE.

Gender		
Boy (n, %)	18 (78 %)	
Girl (n, %)	5 (22 %)	
Age mean (min-max) years	11 (5–17)	
History of atopic diseases (n, %) Asthma Rhinitis Eczema Food allergy Pets Family history of EoE	13 (56.5 %) 2 (8.6 %) 2 (8.6 %) 1 (4.3 %) 1 (6.25 %) 0 (0)	
No atopic history	4 (17.3 %)	
Symptoms at presentation (n, %)		
Dysphagia		9 (39.1 %)
Gastrointestinal reflux symptoms		4 (17.3 %)
Vomiting		10 (43.5 %)

EoE = eosinophilic esophagitis.

### 3.3. Allergy testing and dietary management

All patients underwent specific IgE testing and the Turkish Food Allergy Panel test. An individualized elimination diet was initiated for patients with confirmed food allergies. Two patients showed no detectable sensitization to any allergen (Table 2). Inhalant-specific IgE positivity was found in 2 cases, and wheat-specific IgE positivity was found in 1 case.

**Table 2:** Food allergies in the 23 children with eosinophilic esophagitis.

Food	Patient number
Milk	6, 7
Egg	6, 14, 16
Wheat	9, 16
Peanut	5, 8, 9, 11, 13, 23
Soy	5, 10, 13, 20, 23
Dust allergy	2, 3, 5, 11
Cat allergy	7, 11, 12, 22
Mould allergy	6, 10, 20
Olive tree powder	3, 19, 21
Pollen	4, 13
Chicken	12, 14
Walnut	2, 4, 9, 11, 13
Kidney bean	4, 12
Tomatoes	11, 13
Kiwi	8, 10, 17
Cherry	1
Onion	1
Sweetcorn	4, 8
Apple	6, 8
Strawberry	8, 19
Peach	1, 8, 10
Melon	11
Potatoes	14
Grape	1, 8
Mulberry	1
Pumpkin seeds	4
Apricot	1, 19, 21

No allergy was detected in cases number 15 and 18.

### 3.4. Radiological and endoscopic findings

Prior to endoscopy, esophagogastroduodenal radiography was performed in 22 patients (95.6%), revealing distal esophageal narrowing in 4 patients (17.3%). In these cases, the initial endoscopy was conducted using a 5.6 mm diameter pediatric endoscope. In follow-up procedures, a standard 11.6 mm endoscope was used in 3 cases, whereas 1 case required a repeat procedure with a smaller scope due to persistent luminal narrowing. One patient with a severe stricture was referred to a specialized center for dilation therapy. In total, 63 endoscopic procedures were performed in 22 patients.

### 3.5. Endoscopic evaluation

Endoscopic findings were evaluated using the EREFS. Normal mucosa was observed in 3 patients. Among the remaining cases, varying degrees of edema (stage 1 in 1 case, stage 2 in 9 cases), rings (stage 1 in 2 cases, stage 2 in 4 cases, stage 3 in 1 case), exudates (stage 1 in 2 cases), and furrowing (1 case) were documented (Table 3). In the subgroup of 9 patients presenting with dysphagia, EREFS revealed severe grade 2 furrows and exudates in 2 cases, grade 1 tracheization in 1 case, grade 2 tracheization in 4 cases, and grade 3 tracheization in 1 case. In contrast, among the 14 patients with vomiting and symptoms consistent with gastroesophageal reflux, mild mucosal changes were identified, including grade 1 and grade 2 edema, with 3 cases showing normal mucosa. The most severe case was referred for advanced intervention (4.34%).

**Table 3:** First endoscopic reference scores of the cases.

	n (%)
Normal mucosa	3
Edema (loss of vascular markings) Grade 0: Distinct vascularity Grade 1: Decreased Grade 2: Absent	– 1 9
Rings (trachealization) Grade 0: None Grade 1: Mild (ridges) Grade 2: Moderate (distinct rings) Grade 3: Severe (not pass scope)	– 2 4 1
Exudate (white plaques) Grade 0: None Grade 1: Mild (<10% surface area) Grade 2: Severe (>10% surface area)	– 2 –
Furrows (vertical lines) Grade 0: None Grade 1: Mild Grade 2: Severe (deep)	– – 1
Stricture Grade 0: Absent Grade 1: Present	– –

### 3.6. Treatment and outcomes

Combination therapy involving PPIs, targeted elimination diet, and topical corticosteroids was administered to 22 patients. Two patients without identifiable allergies were placed on an empirical milk and egg elimination diet.

At the 6-month follow-up, adherence to the prescribed diet was deemed satisfactory in all 20 patients on targeted elimination and in both patients on milk–egg exclusion. Complete symptom resolution was achieved in all 22 cases, and histological evaluation during follow-up endoscopy showed resolution of eosinophilic infiltration (95.6%).

A comparative summary of the PEESS and EREFS scores before and after treatment is presented in Table 4. Parallel improvements in both symptom scores and endoscopic findings were observed in most patients. Post-treatment EREFS could not be assessed in 1 patient because of referral for dilation therapy.

**Table 4:** Comparison of PEESS and EREFS scores before and after treatment in 23 pediatric patients with eosinophilic esophagitis.

Patient no.	PEESS pre-treatment	PEESS post-treatment	EREFS pre-treatment	EREFS post-treatment
1	18	4	7	1
2	22	5	8	2
3	20	6	6	2
4	16	3	7	1
5	19	7	6	2
6	21	5	9	2
7	24	6	7	3
8	23	5	8	1
9	17	4	6	2
10	18	5	7	2
11	20	6	7	2
12	22	7	6	3
13	19	5	7	1
14	23	6	8	2
15	21	5	7	1
16	24	6	9	2
17	20	5	6	1
18	19	4	7	1
19	22	5	8	2
20	18	3	6	1
21	21	5	7	2
22	20	6	6	1
23	23	5	8	Not Available

EREFS *=* Eosinophilic Esophagitis Reference Score, PEESS *=* Pediatric Eosinophilic Esophagitis Symptom Score.

### 3.7. EUS findings

**Figure 1.**
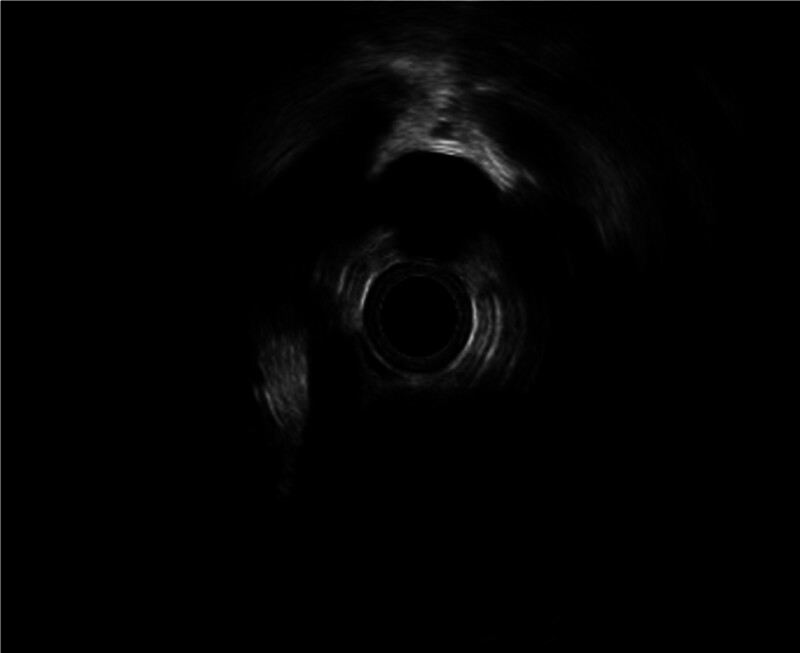
The esophageal wall thickness was normal and was measured as 1.5 mm.

**Figure 2.**
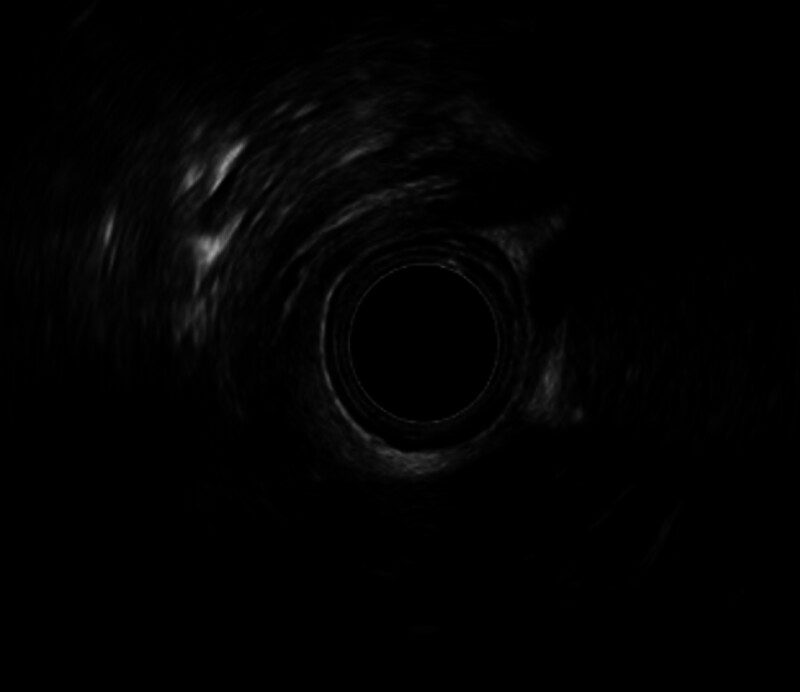
Proximal esophageal wall thickness was measured as 2.2 mm.

EUS was performed in 8 patients during follow-up. Six of these patients demonstrated stage 2 edema at diagnosis. Despite normalization of the mucosal appearance, EUS revealed increased proximal esophageal wall thickness ranging from 1.9 to 2.1 mm. In 1 patient with initial stage 1 edema, the wall thickness remained within normal limits (1.5 mm) (Fig. 1). Another patient, followed up for 4 years and managed exclusively with a tailored diet for the past 2 years, presented with persistent endoscopic ring formation; however, histological remission was noted despite a wall thickness of 2.2 mm on EUS (Fig. 2). A detailed summary of the EUS findings in these 8 patients is presented in Table 5.

**Table 5:** Summary of EUS findings in 8 pediatric patients with eosinophilic esophagitis.

Patient no.	Initial EREFS edema grade	Endoscopic appearance at follow-up	EUS wall thickness (mm)	Histological remission
1	2	Normal	2.0	Yes
2	2	Normal	2.1	Yes
3	2	Normal	1.9	Yes
4	2	Normal	2.0	Yes
5	2	Normal	2.0	Yes
6	2	Normal	2.1	Yes
7	1	Normal	1.5	Yes
8	NA	Persistent rings	2.2	Yes

Initial edema grade not documented for this patient.

EREFS *=* Eosinophilic Esophagitis Reference Score, EUS *=* Endoscopic Ultrasonography, NA *=* not available.

### 3.8. Steroid discontinuation and long-term follow-up

In 10 patients who received both topical corticosteroids and dietary therapy, corticosteroids were withdrawn following initial improvement, and dietary management alone was continued. Following the discontinuation of steroid therapy, patients were monitored exclusively with personalized elimination diets for a mean duration of 14 months (range: 6–24 months); during this period, no clinical relapse or histological recurrence was observed in any of the patients.

## 4. Discussion

Eosinophilic esophagitis can occur at any age, but the emergence of symptoms may be gradual. In this study, the median age at diagnosis was 11 years (range, 5–17 years), and consistent with the literature, the youngest diagnosed patient was 5 years old.^[14]^

In younger children, symptoms typically present as gastroesophageal reflux-like manifestations, such as vomiting, whereas in older children and adolescents, dysphagia and food impaction are the most frequently observed symptoms.^[13]^ In our cohort, 9 patients were aged 15 years or older, and vomiting symptoms were predominantly noted in younger children.

In both pediatric and adult patients diagnosed with EoE, comorbid allergic diseases such as asthma, allergic rhinitis, food allergies, and atopic dermatitis are commonly observed.^[15]^ Similarly, in our study, allergic diseases were present in most cases, with only 4 patients lacking any documented allergy history.

The diagnosis of EoE is established based on clinical symptoms, endoscopic findings, and histopathological evaluation.^[8]^ Prior to endoscopy, radiographs of the esophagus, stomach, and duodenum were obtained for 22 patients. In cases of esophageal narrowing, the diameter of the endoscope was adjusted accordingly before the procedure.

The Endoscopic Reference Score evaluates the severity of 5 key endoscopic features: edema, esophageal rings, white plaques (exudates), linear furrows, and strictures. In a retrospective study involving pediatric patients undergoing diagnostic or follow-up endoscopy, EREFS was effective in identifying EoE.^[11]^ In this study, both clinical symptoms and endoscopic findings were evaluated using the validated scoring systems, PEESS and EREFS, respectively. PEESS enabled standardized assessment of symptom severity before treatment and during follow-up, while EREFS facilitated objective grading of endoscopic changes. Although not statistically correlated owing to the small sample size, parallel improvement was observed in both PEESS and EREFS scores in the majority of patients, indicating a consistent therapeutic response. Future studies with larger cohorts may further explore the relationship between symptomatic and endoscopic improvements using these complementary tools.

The management of eosinophilic esophagitis involves both dietary modifications and pharmacological interventions, each with distinct advantages and limitations. The primary therapeutic goal is the complete resolution of symptoms, along with the restoration of both macroscopic and microscopic normalcy in the esophagus. Among the objective markers, histological assessment through absolute eosinophil count remains the most reliable indicator of inflammatory activity.^[16]^ To achieve remission, 3 main dietary strategies have been developed: an amino acid-based formula that eliminates all potential allergens; a selective elimination diet tailored to patient-specific IgE levels, skin prick test, and atopy patch test results; and a broad-spectrum exclusion diet that removes common allergens such as milk, soy, eggs, wheat, peanuts, and seafood.^[17]^

In this study, specific allergens were assessed in detail for each patient. Instead of implementing complex 5- or 6-food elimination diets, personalized dietary plans were applied in 23 cases. This approach demonstrated that high treatment adherence and effectiveness can be achieved with less restrictive diets. In 2 patients without identifiable allergies, a diet excluding milk and eggs led to significant symptom improvement. All patients strictly adhered to the prescribed dietary regimens, supporting the practicality and effectiveness of personalized dietary interventions.

Topical corticosteroids have proven effective in inducing remission in both pediatric and adult patients with EoE. Although oral corticosteroids are potent, their systemic adverse effects limit their routine use in EoE management^.[13,18]^

The literature highlights the value of high-resolution EUS in pediatric patients diagnosed with eosinophilic “allergic” esophagitis. EUS has been shown to be a sensitive modality for evaluating esophageal inflammation and structural alterations.^[10]^ Notably, its ability to detect histopathological changes, such as mucosal and submucosal thickening, emphasizes its role in both the diagnosis and assessment of the therapeutic response.

This study has several limitations. First, the retrospective design may have introduced selection bias and uncontrolled confounding variables. Second, the relatively small sample size limits the statistical power and generalizability of the findings. Another limitation of the study is the absence of a control group, such as patients with gastroesophageal reflux disease or other esophageal disorders, which could have enhanced the specificity of the findings.

In this context, broader clinical implementation of EUS may offer significant diagnostic advantages, particularly in cases where endoscopic appearance is normal and eosinophilic infiltration is suspected. EUS was performed in only 8 patients due to limited availability and clinical indications. Although EUS provides valuable insights into esophageal wall thickening, the small sample size limits the generalizability of these findings. Large-scale studies are required to validate their routine use.

In addition to histological and radiological assessments, this study utilized 2 validated scoring systems, PEESS for clinical symptoms and EREFS for endoscopic findings, to objectively monitor treatment response. The consistency observed between improvements in the PEESS and EREFS scores supports their complementary use in follow-up evaluations. Notably, in a patient who could not undergo follow-up endoscopy due to an esophageal stricture requiring dilation, PEESS served as a reliable surrogate to assess clinical improvement. These tools may be especially valuable in pediatric patients where repeated endoscopy is not always feasible. Similar findings have been reported in previous studies, emphasizing the utility of PEESS and EREFS in pediatric EoE management and clinical decision-making^.[11,12]^

To our knowledge, this is one of the first pediatric studies to combine PEESS, EREFS, and EUS to monitor the EoE response.

## 5. Conclusion

This study highlights the effectiveness of a multidisciplinary approach in the management of pediatric eosinophilic esophagitis. The combined use of clinical symptom scores, endoscopic findings, histological evaluation, and EUS provides a more reliable and comprehensive assessment of disease activity. In particular, the consistent improvements observed in both the PEESS and EREFS scores support their complementary role in guiding clinical decision-making. Moreover, achieving histological remission in 95.6% of patients through an individualized elimination diet combined with corticosteroid therapy demonstrates that less restrictive, yet effective, treatment strategies can be successful. These findings suggest that EoE can be managed using approaches that enhance treatment outcomes and improve patient comfort, offering valuable insights for future large-scale studies. Further prospective, multi-center studies are warranted to confirm these findings and better define the role of EUS in monitoring disease activity and subepithelial changes in pediatric EoE. Although the mean follow-up period of 14 months allowed for evaluation of short- to mid-term treatment responses, future studies should incorporate longer follow-up durations to assess the sustainability of remission and potential late complications.

## Author contributions

**Conceptualization:** Gül Çirkin.

**Data curation:** Gül Çirkin.

**Formal analysis:** Gül Çirkin, Suna Asilsoy.

**Funding acquisition:** Özge Atay.

**Investigation:** Yeşim Öztürk.

**Methodology:** Yeşim Öztürk.

**Project administration:** Yunus Güler, Yeşim Öztürk.

**Resources:** Yunus Güler, Özlem Gülpinar Aydin.

**Software:** Özlem Gülpinar Aydin, Özge Kangalli Boyacioğlu.

**Supervision:** Yeşim Öztürk.

**Validation:** Gül Çirkin, Özge Atay, Özge Kangalli Boyacioğlu.

**Visualization:** Gül Çirkin, Suna Asilsoy, Nevin Uzuner.

**Writing – original draft:** Gül Çirkin, Yeşim Öztürk.

**Writing – review & editing:** Gül Çirkin, Yeşim Öztürk.

## References

[1] DellonESHiranoI. Epidemiology and natural history of eosinophilic esophagitis. Gastroenterology. 2018;154:319–32.e3.28774845 10.1053/j.gastro.2017.06.067PMC5794619

[2] BhesaniaNSelvakumarPKCPatelS. Eosinophilic esophagitis: a review of the pediatric population and consideration of upcoming therapies. J Gastroenterol Hepatol. 2022;37:420–7.34655451 10.1111/jgh.15706

[3] Van RhijnBDVerheijJSmoutAJBredenoordAJ. Rapidly increasing incidence of eosinophilic esophagitis in a large cohort. Neurogastroenterol Motil. 2013;25:47–52.e5.22963642 10.1111/nmo.12009

[4] StraumannASchoepferA. Update on basic and clinical aspects of eosinophilic oesophagitis. Gut. 2014;63:1355–63.24700438 10.1136/gutjnl-2013-306414

[5] DellonESKimHPSperrySLWRybnicekDAWoosleyJTShaheenNJ. A phenotypic analysis shows that eosinophilic esophagitis is a progressive fibrostenotic disease. Gastrointest Endosc. 2014;79:577–85.e4.24275329 10.1016/j.gie.2013.10.027PMC4599711

[6] WarnersMJNijhuisRABODe WijkersloothLRHSmoutAJPMBredenoordAJ. The natural course of eosinophilic esophagitis and long-term consequences of undiagnosed disease in a large cohort. Am J Gastroenterol. 2018;113:836–44.29700481 10.1038/s41395-018-0052-5

[7] SchoepferAMSafroneevaEBussmannC. Delay in diagnosis of eosinophilic esophagitis increases risk for stricture formation in a time-dependent manner. Gastroenterology. 2013;145:1230–6.e1.23954315 10.1053/j.gastro.2013.08.015

[8] LiacourasCAFurutaGTHiranoI. Eosinophilic esophagitis: Updated consensus recommendations for children and adults. J Allergy Clin Immunol. 2011;128:3–20.e6; quiz 21.21477849 10.1016/j.jaci.2011.02.040

[9] DeBrosseCWCollinsMHButzBKB. Identification, epidemiology, and chronicity of pediatric esophageal eosinophilia, 1982–1999. J Allergy Clin Immunol. 2010;126:112–9.20620567 10.1016/j.jaci.2010.05.027PMC4115587

[10] PytrusTAkutkoKKofla-DłubaczAStawarskiA. Endoscopic ultrasonography in children with eosinophilic esophagitis – a review. Pediatr Rep. 2022;14:13–9.35076585 10.3390/pediatric14010003PMC8788551

[11] WechslerJBBoltonSMAmsdenKWershilBKHiranoIKagalwallaAF. Eosinophilic esophagitis reference score accurately identifies disease activity and treatment effects in children. Clin Gastroenterol Hepatol. 2018;16:1056–63.29248734 10.1016/j.cgh.2017.12.019PMC6003847

[12] MartinLJFranciosiJPCollinsMH. Pediatric Eosinophilic Esophagitis Symptom Scores (PEESS v2.0) identify histologic and molecular correlates of the key clinical features of disease. J Allergy Clin Immunol. 2015;135:1519–28.e8.26051952 10.1016/j.jaci.2015.03.004PMC4460579

[13] Lucendo AJ Molina-Infante J Arias A. Guidelines on eosinophilic esophagitis: evidence-based statements and recommendations for diagnosis and management in children and adults. United Eur Gastroenterol J. 2017;5:335–58. 10.1177/2050640616689525 PMC5415218 28507746

[14] CavalliEBrusaferroAPieriES. Eosinophilic esophagitis in children: doubts and future perspectives. J Transl Med. 2019;17:250.31399124 10.1186/s12967-019-2014-0PMC6688237

[15] MarkowitzJEClaytonSB. Eosinophilic esophagitis in children and adults. Gastrointest Endosc Clin N Am. 2018;28:59–75.29129300 10.1016/j.giec.2017.07.004

[16] RothenbergMEAcevesSBonisPA. Working with the US Food and drug administration: progress and timelines in understanding and treating patients with eosinophilic esophagitis. J Allergy Clin Immunol. 2012;130:617–9.22935588 10.1016/j.jaci.2012.06.051PMC3828675

[17] KagalwallaAFSentongoTARitzS. Effect of six-food elimination diet on clinical and histologic outcomes in eosinophilic esophagitis. Clin Gastroenterol Hepatol. 2006;4:1097–102.16860614 10.1016/j.cgh.2006.05.026

[18] NoelRJPutnamPECollinsMH. Clinical and immunopathologic effects of swallowed fluticasone for eosinophilic esophagitis. Clin Gastroenterol Hepatol. 2004;2:568–75.15224281 10.1016/s1542-3565(04)00240-x

